# Extra-hepatic manifestations associated with hepatitis E virus infection: a comprehensive review of the literature

**DOI:** 10.1093/gastro/gov042

**Published:** 2015-09-10

**Authors:** Fateh Bazerbachi, Samir Haffar, Sushil K Garg, John R Lake

**Affiliations:** ^1^Division of Gastroenterology and Hepatology, University of Minnesota, Minneapolis, MN 55455, USA; ^2^Department of Gastroenterology and Hepatology, University of Damascus, Damascus, Syrian Arab Republic

**Keywords:** hepatitis E virus, viral hepatitis, extra-hepatic manifestations, vaccine, primary prevention

## Abstract

**Background and aims:** Hepatitis E virus (HEV) infection is a significant public health problem that afflicts almost 20 million individuals annually and causes acute liver injury in 3.5 million, with approximately 56 000 deaths. As with other viral hepatitides, extra-hepatic manifestations could represent an important aspect of this infection. The spectrum of these manifestations is still emerging. Acute pancreatitis and neurological, musculoskeletal, hematological, renal, and other immune-mediated manifestations have been described. The aim of this article is to comprehensively review the published literature of extra-hepatic manifestations associated with HEV infection.

**Data sources:** We searched the PubMed database using the MeSH term “hepatitis E” and each of the extra-hepatic manifestations associated with HEV infection. No language or date restrictions were set in these searches. Searches retrieving articles with non-A, non-B hepatitis were excluded. Additional articles were identified through the reference lists of included articles.

**Results:** Several extra-hepatic manifestations associated with HEV infection have been published. The temporal association between some extra-hepatic manifestations and HEV infection and the exclusion of other possible etiologies suggests that HEV infection could have caused some of them. According to the available data, HEV infection appears to be strongly associated with acute pancreatitis, neurological disorders (with primarily dominant peripheral nerve involvement, most commonly manifested as Guillain-Barré syndrome, followed by neuralgic amyotrophy), hematological diseases (hemolytic anemia due to glucose phosphate dehydrogenase deficiency, and severe thrombocytopenia), glomerulonephritis, and mixed cryoglobulinemia. More data are needed to clarify whether an association exists with musculoskeletal or other immune-mediated manifestations.

**Conclusions:** HEV infection should be considered in patients with acute pancreatitis, Guillain-Barré syndrome, neuralgic amyotrophy, hemolytic anemia due to glucose phosphate dehydrogenase deficiency, severe thrombocytopenia, glomerulonephritis, and mixed cryoglobulinemia. Alternatively, signs and symptoms of these conditions should be sought in patients with acute or chronic HEV infection. More data are needed to confirm the role of HEV in other extra-hepatic disorders.

## Introduction

Hepatitis E virus (HEV) infection is an important public health problem in the developing world and presents two major issues. According to the fact sheet on hepatitis E, published by the World Health Organization (WHO) and updated in June 2014, there are each year 20 million hepatitis E infections, over 3 million acute cases of hepatitis E, and 56 600 hepatitis E-related deaths, with the highest prevalence in East- and southern Asia [1]. In the developed countries, hepatitis E infection is an emerging disease. It was traditionally thought to occur in individuals travelling to areas where the disease is endemic; however, cases of sporadic autochthonous hepatitis E have been reported in individuals with no history of recent travel [2]. In a recent analysis of the National Health and Nutrition Evaluation Survey (NHANES), the seroprevalence of hepatitis E in the USA was estimated at 6%, despite the rarity of reported cases of hepatitis E [3]. Its seroprevalence in blood donors in the United Kingdom is estimated to be 11% [4]. In the Toulouse region in south-west France—an area in which the disease is considered to be hyperendemic—the seroprevalence in blood donors was initially thought to be 16%, but rose to 52% using more sensitive assays [5].

HEV can cause asymptomatic, icteric, or fulminant acute hepatitis [6]. Cases of chronic hepatitis E were first reported in 2008 [7]. Chronic hepatitis has been described in HEV genotype 3 infection, which occurs in western Europe and North America, almost exclusively among immunosuppressed individuals. Some patients with chronic hepatitis E develop progressive liver disease, resulting in advanced fibrosis or cirrhosis [8].

In immunocompetent individuals, acute hepatitis E is diagnosed, based on the detection of anti-HEV immunoglobulin M (IgM), increased titers of anti-HEV immunoglobulin G (IgG), or detection of HEV RNA in blood or stool. In immunocompromised individuals, acute hepatitis E is diagnosed, based on detection of HEV RNA in blood or stool [9]. Given that serological techniques vary in their accuracy [10], some authors recommend confirming serologically-detected cases with molecular techniques (HEV RNA) [11]. Chronic hepatitis E is defined by persistent HEV replication for more than 6 months, or more than 3 months in the setting of organ transplantation [12].

The number of papers published on HEV infection and indexed in PubMed has increased substantially during the last decade. Numerous extra-hepatic manifestations are reported in association with acute or chronic hepatitis E [13]. Although causality is uncertain [14], the temporal association between HEV infection and the extra-hepatic manifestations, plus the exclusion of other possible etiologies suggest that HEV infection may be causal. These extra-hepatic manifestations can overshadow the hepatic injury and HEV may not be suspected. The aim of this article is to comprehensively review the published literature on extra-hepatic manifestations associated with HEV infection.

## Search strategy and selection criteria

Two independent investigators (FB and SH) searched PubMed on March 25, 2015 using the MeSH term “hepatitis E” AND each of the following MeSH terms: “pancreatitis”, “Guillain-Barre syndrome”, “brachial plexus neuritis”, “peripheral nervous system diseases”, “meningitis”, “encephalitis”, “myelitis”, “myositis”, “arthralgia”, “glucosephosphate dehydrogenase deficiency”, “anemia”, “thrombocytopenia”, “agranulocytosis”, “macrophage activation syndrome”, “monoclonal gammopathy”, “glomerulonephritis”, “renal insufficiency”, “cryoglobulinemia”, “Schöenlein-Henoch purpura”, “myasthenia gravis”, “thyroiditis”, “hyperthyroidism”, and “myocarditis”. No language or date restrictions were set in these searches. Searches retrieving articles with non-A, non-B hepatitis were excluded. Additional articles were sourced manually by searching the bibliographies of relevant articles. The extra-hepatic manifestations associated with HEV infection found in these searches were classified into different categories as shown in Table 1. There were no disagreements between the two investigators regarding the search results. An example of inter-investigator discussion was the choice of acute pancreatitis classification (mild, moderately severe, or severe) based on Revision of the Atlanta classification. Another example was discussion of duplicated cases of neurological manifestations and making sure that no duplicates were added to the manuscript.

**Table 1.** Extra-hepatic manifestations of HEV infection

Manifestation	Type	
Acute pancreatitis		
Neurological	Central nervous system diseases	Meningitis **-** encephalitis **-** meningoencephalitis Ataxia Pyramidal syndrome Pseudotumor cerebri Acute transverse myelitis
	Peripheral nervous system diseases	Guillian-Barre syndrome Neuralgic amyotrophy Cranial nerve diseases: Bell palsy **-** oculomotor palsy
Musculoskeletal	Necrotizing myositis Pyomyositis	
Hematological	Hemolytic anemia Aplastic anemia Pure red-cell aplasia Severe thrombocytopenia Hemophagocytic syndrome MGUS	G6PD deficiency Auto-immune
Renal	Decreased eGFR Glomerulonephritis ± cryoglobulinemia	Membranous glomerulonephritis Membranoproliferative glomerulonephritis IgA nephropathy Nephroangiosclerosis
Other immune-mediated	Thyroiditis Myocarditis Henoch–Schönlein purpura Myasthenia gravis	

eGFR = estimated glomerular filtration rate; G6PD = glucose-6-phosphate dehydrogenase; MGUS = monoclonal gammopathy of uncertain significance

## Acute pancreatitis

Acute pancreatitis (AP) in the setting of fulminant viral hepatitis is well recognized, and mortality depends on the severity of hepatitis, rather than pancreatitis [15, 16]. AP is rarely associated with non-fulminant viral hepatitis. Most frequently, these cases are attributed to hepatitis A virus (HAV), hepatitis B virus (HBV) or hepatitis C virus (HCV) [17]. The first documented case of non-fulminant HEV-associated AP was reported in 1999 by Mishra *et al*.** [18], along with five other cases of HAV-related AP. To the best of our knowledge, at least 13 other case reports [19–31] and 4 case series [32–35] have been reported, with a total of 56 patients (Table 2). One study was in the form of an abstract [34], and all others were original articles. Two of the case series were prospective [33, 35], and the other two were retrospective [32, 34]. Three patients were excluded: one patient had other plausible causes of AP (including medications) [29], and two patients had fulminant acute hepatitis E according to the criteria in the position paper on acute liver failure, published by the American Association for the study of Liver Diseases [34–36]. All cases fulfilled the criteria of the American College of Gastroenterology for the diagnosis of AP [37].

**Table 2.** Acute pancreatitis (AP) associated with non-fulminant acute hepatitis E

Authors/year	Country	No. of cases	Age/ sex	HEV diagnosis /genotype	Days to AP^a^	Days in hospital for AP^b^	Severity of AP	Treatment of AP	Outcome
**Case report**									
Mishra 1999 [18]	India	1	14/M	IgM/NT	10	4	Mild	Supportive	Recovery
Majumder 1999 [25]	India	1	32/M	IgM/NT	15	NM	Moderately severe	Surgery	Recovery
Borgohain 2000 [19]	India	1	18/M	IgG**-**IgM/NT	0	9	Mild	Supportive	Recovery
Maity 2002 [24]	India	1	18/M	IgM/NT	30	35	Severe	Hemodialysis	Recovery
Makharia 2003 [26]	India	1	45/M	IgM/NT	0	NM	Mild	Supportive	Recovery
Jaroszewicz 2005 [21] ^c^	Poland	1	28/M	IgM/NT	15	6	Mild	Supportive	Recovery
Thapa 2009 [31] ^d^	India	1	7/M	IgM/NT	12	29	Mild	Supportive	Recovery
Somani 2009 [30]	India	1	35/M	IgM/NT	7	20	Severe	Hemodialysis	Death
Deniel 2011 [20] ^e^	France	1	26/M	IgM-PCR/1a	21	NM	Moderately severe	Supportive	Recovery
Javid 2012 [22]	India	1	36/M	IgG**-**IgM/NT	7	5	Mild	Supportive	Recovery
Rudrajit 2013 [28]	India	1	24/M	IgM/NT	16	6	Mild	Supportive	Recovery
Nayak 2013 [27]	India	1	16/M	IgM-PCR/NT	8	9	mild	Supportive	Recovery
Karanth 2014 [23]	India	1	27/M	IgG**-**IgM**-**PCR/NT	28	14	severe	Supportive	Recovery
**Case series**									
Jain 2007 [33]	India	4	mean 26	IgM/NT	4 (2**–**5)	6 (3**–**12)	4 mild	4 supportive	Recovery
			4 men						
Bhagat 2008 [32]	India	4	mean 21	IgM/NT	15 (12**–**17)	12 (7**–**23)	2 mild	3 supportive	Recovery
			3M/1F				2 moderately severe	1 drainage	
Sudhamshu 2011 [35]	Nepal	17	NM	IgM/NT	NM	NM	16 mild/moderate	1 hemodialysis	1 death
							1 severe	16 supportive	16 recovery
Mohindra 2013 [34]	India	15	mean 25	IgM/NT	8 (0**–**35)	7 (2**–**30)	5 mild	15 supportive	15 recovery
			14M/1F				5 moderately severe		
							5 severe		
**Total**	51 southern Asia	53	mean 24.6 35M/2F	49 IgM	10 (0**–**35)	9 (2**–**35)	44 mild/moderate	47 supportive	2 deaths
	1 France			1 IgG + IgM			9 severe	2 surgery	51 recovery
	1 Poland			3 IgM + PCR					

^a^Days between jaundice and acute pancreatitis

^b^Days in hospital after diagnosis of AP

^c^Indian patient living in Poland with recent travel to India

^d^G6PD deficiency patient

^e^Pakistani-French patient living in France with recent travel to Pakistan

NM=not mentioned; NT=not tested

Of the 53 included patients, 51 were from southern Asia (India and Nepal) and 2 were from western countries, although both had recently travelled to southern Asia [20, 21]. The mean age of patients at diagnosis was 24.6 years (range 7–54) with a male-to-female ratio of 18:1. The diagnosis of acute HEV was based on the presence of anti-HEV IgM in 49 patients, anti-HEV IgG and anti-HEV IgM in 1 patient, and anti-HEV IgM and HEV RNA in 3 patients. Genotyping was performed in one patient and revealed type 1a [20]. It is presumed that all other patients were infected with genotype 1, which is prevalent in that area; it is possible that genotype 1 has high tropism for the pancreas. So far, no cases of AP have been reported in patients infected with other HEV genotypes. Although the presumption that all these patients were infected with genotype 1 may be justified, there is also a possibility that other genotypes may be associated with AP; however—as is the case of fulminant acute hepatitis E, which has been increasingly reported in western Europe, where genotype 3 is prevalent—AP related to acute hepatitis E may occur in these regions as well.

The mean interval between jaundice and AP-pain was 10 days (range 0–35). The mean hospital stay for AP was 9 days (range 2–35). We retrospectively classified these AP cases into mild, moderately severe, or severe, based on Revision of the Atlanta Classification [38], as it is currently the most widely accepted set of criteria. AP was mild or moderately severe in 44 patients (83%) and severe in 9 patients (17%). Mild pancreatitis was not evaluated in a major prospective study of these cases [35], which may have resulted in a selection bias favoring the diagnosis of more severe cases. The overall mortality rate was 3.8% (2 of 53 patients) which is similar to the mortality rate observed for all other causes of AP.

The typical profile is a 25-year-old male residing in southern Asia, developing acute pancreatitis 10 days after the onset of jaundice, usually resolving with supportive treatment, with greater severity than previously thought, but with a similar mortality rate to other causes of AP.

Severe abdominal pain early in the course of acute hepatitis E should alert the clinician to the possibility of associated AP. Early diagnosis of AP complicating acute hepatitis E may help in reducing morbidity and mortality. Despite the rarity of the association between AP and non-fulminant acute hepatitis E, HEV infection should be added to the potential etiologies of AP in areas where the disease is endemic.

## Neurological manifestations of HEV

Neurological manifestations of HEV infection were first reported by Soud in 2000 [39]. We are aware of 42 subsequent reports, involving a total of 77 patients, from regions where the disease is endemic and others where it is not, of neurological manifestations associated with HEV infection [40–81] (Table 3).

**Table 3.** Neurological manifestations associated with HEV infection

Authors/year	Country	No. of cases	Age/sex	Neurological manifestations	Delay hepatitis neurological disorders	IgM	HEV RNA	HEV genotype	ALT (IU/L)	Treatment	Recovery/delay
**Acute hepatitis E – Peripheral manifestations**											
Sood 2000 [39]	India	1	50/M	GBS	5 days	+	NT	NT	114	Supportive	Full/1 month
Kumar 2002 [45]	India	1	35/M	GBS	17 days	+	NT	NT	752	MV/IVIG	Full/2 weeks
Kamani 2005 [46]	India	1	58/F	GBS	9 days	+	NT	NT	1448	IVIG/PP	Full/12 days
Khanam 2008 [47]	Bangladesh	1	20/M	GBS	10 days	+	NT	NT	2509	MV	Full/12 days
Loly 2009 [48]	Belgium	1	66/M	GBS GM2+	Few days	+	NT	NT	1813	IVIG	Full/3 months
Chalupa 2010 [49]	Czech Rep	1	65/M	GBS	No delay for all	+	NT	NT	1600	IVIG	Full/4 months
Kamar 2011 [40]	France	1	60/F	GBS	Concomitant	+	Serum+CSF–	3f	384	IVIG	Partial/18 months
Cronin 2011 [50]	Ireland	1	40/M	GBS GM2+	Concomitant	+	NT	NT	57	MV/IVIG/PP	Full/6 months
Maurissen 2012 [51]	Belgium	1	51/F	GBS GM1 & 2+	Concomitant	+	Serum+	NT	2074	IVIG	Full/1 week
Tse 2012 [52]	Hong Kong	1	60/F	GBS	3 days	+	NT	NT	2858	PP	Full/1 month
Del Bello 2012 [53]	France	1	65/M	GBS, severe myositis	Concomitant	+	Serum+	3f	2000	MV/IVIM/Riba	partial/1 month
Santos 2013 [54]	Portugal	1	58/M	GBS	17 days	+	Serum+	3a	2320	IVIG/MV	partial/2 months
Sharma 2013 [55]	India	1	27/M	GBS	40 days	+	NT	NT	NM	IVIG	Full/NM
Geurtsvan-Kessel 2013 [56]	Bangladesh	11	24/NM	GBS	NM	+	Serum+ in 1	1 in 1	NM	NM	NM
Scharn 2014 [57]	Germany	1	50/M	GBS GM1 & 1B+	Concomitant	+	Serum+CSF–	3c	334	IVIG	Full/5 months
van den Berg 2014 [42]	Netherlands	10	Mean: 54 6M/4F	GBS	Mean: 5 days	+	Serum+ in 3 CSF– in 5	3	mild	NM	NM
Chen 2014 [58]	China	1	64/M	GBS GM2+, encephalitis	5 days	+	NT	NT	1461	MV/IVIG	Full/12 months
Comont 2014 [59]	France	1	73/M	GBS	Concomitant	+	Serum+CSF+	3f	822	IVIG	Full/2 months
Fong 2009 [60]	UK	1	53/M	Bilateral NA	Concomitant	+	NT	NT	2547	Physiotherapy	Full/2 years
Rianthavorn 2010 [61]	Thailand	1	49/M	Bilateral NA	3 days	+	Serum+	3f	795	NM	partial/4 months
Kamar 2011 [40]	UK	1	38/M	Bilateral NA	5 days	+	Serum+	3e	1160	Supportive	partial/18 months
Carli 2012 [62]	France	1	30/M	Left NA	Concomitant	+	NT	NT	1518	Steroid	Full/slow
Inghilleri 2012 [63]	France	1	28/M	Bilateral NA	Concomitant	+	NT	NT	1007	NM	NM
Cheung 2012 [64]	UK	1	56/M	Bilateral NA	Concomitant	+	NT	NT	300	NM	Partial/10 months
Motte 2014 [65]	France	1	52/M	Bilateral NA	7 days	+	Serum+	3f	590	NM	Partial/2 months
Moisset 2014 [66]	France	1	36/M	Bilateral NA	7 days	+	Serum+	3f	1707	IVIG/Riba	Partial/6 months
Deroux 2014 [67]	France	1	38/M	Left NA	Concomitant	+	NT	NT	1612	NM	Partial/4 months
van Eijk 2014 [43]	Netherlands & UK	5	36^1 ^4M/1F	Bilateral NA	NM	+	Serum+ in 4	3 in 1 pt	34–313	NM	Partial in 5/6 months
Woolson 2014 [41]	UK	1	38/M	Bilateral NA	NM	+	Serum +	3	319	Supportive	Partial/12 months
	UK	1	39/M	Bilateral NA	NM	+	Serum +	NT	27	Supportive	Partial/12 months
Theochari 2015 [68]	UK	1	65/M	Bilateral NA	Concomitant	+	NT	NT	1368	Prednisolone	Full/10 months
Décard 2015 [69]	Switzerland	1	47/M	Bilateral NA	Concomitant	+	NT	NT	106	Physiotherapy	Partial/12 months
Kamar 2011 [40]	UK	1	42/M	PRN	Concomitant	+	Serum+CSF-	3e	623	NM	Full/3 months
Despierres 2011 [70]	France	1	49/M	PRN	Concomitant	+	Serum+	3	78	Supportive	Full/2 weeks
Peri 2013 [71]	Italy	1	53/M	PRN	Concomitant	+	Serum+Stool+	3	1768	Supportive	Full/3 months
Yadav 2002 [72]	India	1	13/F	Oculomotor palsy	3 days	+	NT	NT	382	Supportive	Minimal
Dixit 2006 [73]	India	1	32/M	Bell’s palsy	7 days	+	NT	NT	1000	Supportive	Full/3 weeks
Jha 2012 [74]	India	1	28/M	Bell’s palsy	10 days	+	NT	NT	1200	Physiotherapy	Full/3 weeks
Woolson 2014 [41]	UK	1	92/F	Vestibular neuritis	Concomitant	+	Serum+	3	1504	Supportive	Full/7 days
	UK	1	86/M	Neuromyopathy	Concomitant	+	Serum+	NT	285	Supportive	Partial/nm
	UK	1	34/M	Small-fibre neuropathy	2 months	–	Serum+CSF-	NM	–	Gabapentin	No response
Bennett 2015 [75]	UK	1	77/F	Paresthesia	Concomitant	+	Serum+	NT	1606	Supportive	Full/3 weeks
**Acute hepatitis E – Central manifestations**											
Kejariwal 2001 [76]	India	1	28/W	Meningo-encephalitis	Concomitant	+	NT	NT	1890	Supportive	Full/3 weeks
Deroux 2014 [67]	France	1	41/M	Encephalitis	Concomitant	+	Serum+ CSF+	3f	479	Nm	Full/12 weeks
Despierres 2011 [70]	France	1	54/F	Meningitis Diffuse neuralgic pain	Concomitant	+	Serum+ CSF+	3	566	Ceftriaxone/Acyclovir	Full/2 weeks
Naha 2012 [77]	India	1	33/M	Aseptic meningitis	10 days	+	NT	NT	400	Supportive	Full/nm
Mandal 2006 [78]	India	1	12/F	Acute transverse myelitis	20 days	+	NT	NT	NM	Supportive	Full/10 days
Thapa 2009 [79]	India	1	7/M	Pseudotumor cerebri	2 days	+	NT	NT	654	Supportive	Full/3 days
**Chronic hepatitis E – Neurological manifestations**											
Kamar 2010 [44]	France	1	44/M	Pyramidal syndrome, PN	33 months	+	Serum+ CSF+	3f	105	Reduce TAC/ IVIG	Death/3 months
Kamar 2011 [40]	France	1	60/M	Ataxia, confusion, PRN	60 months	+	Serum+ CSF+	3f	171	Change TAC to sirolimus	Partial/10 months
	France	1	35/M	Encephalitis	3 years	+	Serum+ CSF+	3f	110	MV/IVIG, stop IS, foscavir	Full/2 months
	UK	1	48/M	Sensory PN	NM	+	Serum+ CSF+	3a	195	PegIFN/Riba	Full/7 months
Maddukuri 2013 [80]	USA	1	64/M	Ataxia, cognitive decline, PN	12 months	+	Serum+	3	362	Reduce TAC/ PegIFN	Death/48 months
de Vries 2014 [81]	Netherlands	1	66/F	Encephalopathy-ataxia-sensory neuropathy	2 years	+	Serum+CSF+	3	299	Reduce MMF/ Riba	Partial/7 months

ALT=alanine aminotransferase; CSF=cerebrospinal fluid; GBS=Guillain-Barré syndrome; IS = immunosuppressants; IVIG = intravenous immunoglobulin; MMF = mycophenolate mofetil; MV = mechanical ventilation; NA = neuralgic amyotrophy; NM = not mentioned; NT = not tested; PN = peripheral neuropathy; PegIFN = pegylated interferon; PP = plasmapheresis; PRN = polyradiculoneuropathy; Riba = ribavirin; TAC = tacrolimus

Neurological manifestations in patients with HEV infection are uncommon and have been reported as occurring in 7 cases of acute or chronic HEV infection (5.5%) over a 5-year period in a case series from Toulouse, France, and Cornwall, UK [40], and in 8 out of 106 cases of autochthonous acute and chronic hepatitis E (7.5%) over a 14-year period in a recent retrospective study from Cornwall, UK [41]. The spectrum of neurological injury is broad and can be divided into two clinical presentations: the dominant clinical presentation is peripheral nerve involvement—most commonly manifesting as Guillain-Barré syndrome (GBS)—followed by neuralgic amyotrophy (NA); the second and less frequent picture is central involvement in the form of meningitis, encephalitis, meningo-encephalitis, or transverse myelitis. Although only genotype 3 was found among patients in developed countries, cases from southern Asia were not genotyped and many of these cases could have been infected with genotype 1.

Thirty-seven cases of GBS were reported in 16 case reports and 2 case-controlled studies. In these case-controlled studies of GBS of all etiologies—involving 100 patients in Bangladesh and 201 patients in the Netherlands—acute HEV infection was associated with this syndrome in 11% and 5% of patients, respectively [42]. The mean age of the reported patients was 44.5 years (range 20–73) with a male-to-female ratio of 9:4. The mean delay between acute hepatitis E and neurological symptoms was 6 days (range 0–40). In 16 cases for which details of treatment were available, intravenous immunoglobulin (IVIG) was used in 13, mechanical ventilation in 5, plasmapheresis in 3 cases, and ribavirin in 1. Neurological recovery was complete in 13 cases and partial in the remaining three within a period ranging from 1 week to 18 months.

Neuralgic amyotrophy (NA), also known as brachial neuritis or Parsonage-Turner syndrome, is an acute monophasic brachial plexus disorder of unknown cause, although preceding infections have commonly been reported. Eighteen cases of NA were reported in 17 case reports and 1 case series. In this case series of 47 NA patients of all etiologies from the UK and the Netherlands, acute hepatitis E was associated with this neurological disorder in 10% of patients [43]. The mean age of reported patients with NA was 50 years (range 28–65) with a male-to-female ratio of 8:1. The delay between acute hepatitis E and neurological symptoms ranged from 0–7 days. NA was bilateral in 16 cases and unilateral in 2 cases (88%). In eight cases for which details of treatment were available, steroids were used in two, physiotherapy in two, IVIG and ribavirin in one case, and the treatment was supportive in three cases. Neurological recovery was complete in three cases after follow-up periods of 10–24 months, and partial in 14 cases after follow-up periods of 2–24 months.

The finding that hepatitis E is associated with both GBS and NA may suggest that these syndromes reflect differing parts of the same spectrum of neurological immune-mediated diseases [43].

Six cases have been reported of neurological manifestations of chronic hepatitis E following solid organ transplantation (five cases) and HIV infection (one case). HEV RNA has been found in both the serum and the cerebrospinal fluid (CSF) in the five tested patients, which suggests that HEV replication may occur in this compartment. Analysis of such HEV RNA in one patient shows that the variants differed from those observed at the same time point in the serum, which suggests the presence of neurotropic quasispecies [44]. The first-line therapy for chronic HEV in solid organ transplant recipients is to reduce immunosuppressants when possible. The second-line therapy in these patients is the administration of ribavirin. Full neurological recovery following treatment was noted in one patient, partial recovery in two and death, related to decompensated cirrhosis and neurological deterioration, in two. Full neurological recovery was observed in the sixth patient with HIV infection following treatment with peginterferon and ribavirin.

It is recommended that clinicians consider the possibility of HEV infection in patients with neurological disorders and concurrent transaminase elevation, especially those with peripheral nerve involvement. The diagnosis may be suggested by HEV serology but should be confirmed with molecular testing in serum, CSF, or both. The recognition of HEV infection in a patient presenting with neurological manifestations could present an opportunity to treat active HEV infection with antivirals before chronic damage takes place, but further studies are needed to clarify their role in this setting.

## Musculoskeletal manifestations of HEV

Several musculoskeletal manifestations associated with the acute phase of HEV infection have been reported: (i) asymptomatic elevation of creatine phosphokinase (CK) of MM type indicating skeletal muscle damage [82], (ii) acute polyarthritis lasting for 3 months and resolving spontaneously [83], (iii) necrotizing myositis associated with GBS in a liver-transplant patient resolving after ribavirin administration [53], (iv) pyomyositis 4 weeks after recovery from acute hepatitis E in a patient with recent history of type 2 diabetes [84], (v) inflammatory polyarthralgia revealing acute hepatitis E [85], and (vi) arthralgia associated with a diffuse maculopapular rash resolving with supportive measures [86] (Table 4). Further studies are needed to confirm the association of HEV infection with musculoskeletal manifestations.

**Table 4.** Musculoskeletal manifestations associated with acute HEV infection

Authors/year	Country	Age/sex	HEV diagnosis/ genotype	Manifestations	Treatment	Outcome
Kitazawa 2003 [82]	Japan	59/M	IgM/NT	Elevated CK MM type	Supportive	Recovery
Serratrice 2007 [83]	France	51/W	PCR/3	Acute polyarthritis	Supportive	Recovery
Del Bello 2012 [53]	France	65/M	PCR/3f	Necrotizing myositis, GBS	Ribavirin	Recovery
Annamalai 2013 [84]	India	39/M	NM	Pyomyositis	Surgical drainage	Recovery
Bialé 2013 [85]	France	40/F	IgM-PCR/NT	Inflammatory polyarthralgia	Supportive	Recovery
Al-Shukri 2013 [86]	UK	52/F	IgM-PCR/3	Arthralgia, maculopapular rush	supportive	Recovery

CK = creatine phosphokinase; GBS = Guilain-Barré syndrome; NM = not mentioned; NT = not tested

## Hematological manifestations of HEV

### Hemolytic anemia

#### Hemolytic anemia due to glucose-6-phosphate dehydrogenase deficiency

HEV is endemic in southern Asia, which is home to a significant proportion of glucose-6-phosphate dehydrogenase (G6PD)-deficient individuals and instances of co-existence of both the conditions should not be rare; however, only seven case reports [31, 87–92] and three small case series [93–95], with a total of 17 cases, have been published of severe hemolysis occurring in patients with acute hepatitis E associated with G6PD deficiency (Table 5). All cases originated from southern Asia and were thought to be attributed to genotype 1. Patients presented with high-grade fever, chills, neutrophilic leukocytosis, severe hyperbilirubinemia, and renal failure—a combination that is seldom encountered in uncomplicated viral hepatitis. Their serum bilirubin ranged from 28–66 mg/dL. Acute renal failure was present in 10 patients and their serum creatinine ranged from 5.4–9.2 mg/dL, necessitating hemodialysis in seven of them. Death due to cerebral bleeding, sepsis, and hepatic failure was reported in three patients. Given the rarity of published cases, it is quite possible that more severe cases are reported, whereas less severe ones are under-diagnosed and under-reported. Wilson’s disease should be ruled out in this setting, along with other causes of hemolyic anemia. Tests for G6PD deficiency may be negative during and immediately after a hemolytic episode and should be performed 8–10 weeks after the disease subsides. Administration of vitamin K should be avoided in these patients because it may further aggravate hemolysis. Renal failure may be non-oliguric; therefore, kidney function should be assessed by regularly monitoring blood chemistry, urinary sodium and osmolarity. Preventive measures against acute renal failure—such as maintenance of high urinary output, correction of fluid and electrolyte imbalance and avoidance of nephrotoxic drugs—should be implemented early. All cases of acute viral hepatitis with marked hyperbilirubinemia should be observed carefully for impaired renal function and hemolysis. However hemolysis due to G6PD deficiency is not an extrahepatic manifestation in the strict sense. Rather, instigation of hemolysis in G6PD-deficient individuals may be associated with HEV infection, as with several other infections.

**Table 5.** Hemolytic anemia due to G6PD deficiency associated with acute HEV infection

**Authors/year**	Country	Age/sex	HEV diagnosis/ genotype	Hb (g/dL)	Total bilirubin (mg/dL)	Creatinine (mg/dL)	Treatment	Outcome
Abid 2002 [93]	Pakistan	26/M	IgM/NT	6.4	42	7.8	Hemodialysis	Recovery
	Pakistan	16/M	IgM/NT	7.2	32	5.9	Supportive	Recovery
	Pakistan	14/M	IgM/NT	6.2	38	6.4	Supportive	Recovery
	Pakistan	22/M	IgM/NT	6.2	42	9.8	Hemodialysis	Recovery
	Pakistan	35/M	IgM/NT	5.5	28	5.4	Supportive	Recovery
Monga 2003 [87]	India	35/M	IgM/NT	8	48	–	Supportive	Recovery
Zamvar 2005 [88]	Pakistan	10/F	IgM/NT	3.6	44	–	Transfusion	Recovery
Thapa 2009 [89]	India	7/M	IgM/NT	7.0	42	Normal	Supportive	Recovery
Thapa 2009 [31]^a^	India	7/M	IgM/NT	7.6	35	–	Supportive	Recovery
Somani 2011 [91]	India	17/M	IgM/NT	5.5	66	9.2	Hemodialysis	Recovery
Au 2011 [94]^b^	China	54/M	IgM/NT	5.8	50	Renal failure	Transfusion, hemodialysis	Death: cerebral bleeding
Au 2012 [90]	China	53/M	IgM/NT	7.9	–	Renal failure	Transfusion, hemodialysis	Death: sepsis
	China	54/M	IgM/NT	8.3	–	Renal failure	Hemodialysis	Death: hepatic failure
Jain 2013 [95]	India	NM	IgM/NT	NM	–	–	NM	Recovery
	India	NM	IgM/NT	NM	–	–	NM	Recovery
	India	NM	IgM/NT	NM	–	–	NM	Recovery
Tomar 2014 [92]	India	–	IgM/NT	–	–	Renal failure	Supportive, hemodialysis	Recovery

^a^Patient with associated acute pancreatitis

^b^Patient with raised methemoglobinemia

G6PD = glucose-6-phosphate dehydrogenase; NM = not mentioned; NT = not tested

#### Auto-immune hemolytic anemia

Auto-immune hemolytic anemia (AIHA) is rarely associated with viral hepatitis. HCV infection has been the main reported association, but cases of HAV and HBV have been also described [96]. Three documented cases of AIHA associated with HEV infection have been published [97–99]. These cases revealed sudden and rapid drops in hemoglobin levels during the course of illness and were diagnosed after excluding other causes of anemia and hemolysis. Two patients were treated supportively with good outcomes [98, 99], and the treatment administered to the third patient was not mentioned [97].

#### Severe thrombocytopenia

A variety of hepatotropic viruses are known to cause severe thrombocytopenia. HEV infection associated with severe thrombocytopenia has been cited in six case reports [100–105] and one case series [106]—not necessarily in regions where the disease is endemic—with a total of nine cases (Table 6). The HEV genotype was not tested in all reports from southern Asia. Genotype 3 was found in patients originating from regions where HEV is not endemic. The diagnosis relies on the exclusion of other causes of thrombocytopenia. Patients' platelet counts ranged from 1 x 10^9^/L to 21 x 10^9^/L. Most patients improved spontaneously, while others received platelet transfusion, intravenous immunoglobulin (IVIG) and/or corticosteroids. Recovery was observed in all patients and no fatalities were recorded. The mechanism of severe thrombocytopenia is believed to be immune-mediated, and platelet-associated antibodies have been positive in two out of four tested patients. It may be appropriate to perform HEV testing in patients with severe thrombocytopenia associated with elevated liver enzymes, regardless of the patient’s travel history.

**Table 6.** Severe thrombocytopenia associated with acute HEV infection

Authors/year	Age/sex	Country	HEV diagnosis/genotype	ALT (IU/L)	Purpura	Platelets (/mm^3^)	Anti-platelet antibodies	Co-morbidity	Bone marrow aspirate	Treatment	Outcome
Bulang 2000 [100]^a^	48/M	Germany	IgM/NP	800	–	18 000	NT	Sinusitis	NT	Supportive	Recovery
Ali 2001 [101]^b^	38/M	India	IgM/NP	670	+	10 000	Negative	MGN	NT	IVIG, FFP, steroids	Recovery
Singh 2007 [102]	34/M	India	IgM/NP	783	+	13 000	Positive	–	Normocellular	IVIG, platelets	Recovery
Colson 2008 [103]	72/F	France	PCR/3f	1 520	–	9 000	NT	Nimesulide	Normocellular	Supportive	Recovery
								Transient neutropenia			
Thapa 2009 [104]	8/F	India	IgM/NP	1 080	+	21 000	Positive	Normal	Normo-cellular	IVIG	Recovery
Fourquet 2010 [106]	52/M	France	PCR/3f	2 958	–	13 000	NT	–	NT	Supportive	Recovery
	20/M	France	PCR/3f	461	+	1 000	Negative	–	NT	Steroids	Recovery
	61/M	France	PCR/3f	1 598	–	10 000	NT	–	normo-cellular	Supportive	Recovery
Masood 2014 [105]	25/M	Pakistan	IgM/NP	1045	–	9 000	NT	–	normo-cellular	Platelets	Recovery

^a^Indian patient living in Germany with recent travel to India

^b^Patient with membranous glomerulonephritis

FFP = fresh frozen plasma; IVIG = intravenous immunoglobulin; MGN = membranous glomerulonephritis; NT = not tested

Less-severe thrombocytopenia without significant consequences was noted in 12 out of 106 patients (11%) in a recent retrospective study of autochthonous acute and chronic hepatitis E in Cornwall, UK [41]. The lowest platelet count recorded at presentation in this study was 40 x 10^9^/L.

#### Hepatitis-associated aplastic anemia

Hepatitis-associated aplastic anemia (HAAA) is a variant of the aplastic anemia syndrome, in which an acute attack of hepatitis leads to marrow failure and pancytopenia. It was first reported in 1955 and by 1975 more than 200 cases had been described [107–109]. HAV, HBV, HCV, hepatitis D virus (HDV), parvovirus B19, cytomegalovirus, and Epstein-Barr virus (EBV) have been associated with HAAA. Pancytopenia typically occurs 2–3 months after the hepatitis episode, which could be fulminant, acute, or chronic. The development of HAAA is always fatal if not managed promptly and the standard therapy is allogenic bone marrow transplantation from Human leukocyte antigen (HLA)-matched siblings, or immunosuppressive therapy if an appropriate donor is not available [110]. Two case reports of HAAA associated with HEV infection have been published, with a fatal outcome in one case and an absence of response to cyclosporine in the other [111, 112].

#### Pure red cell asplasia

Pure red cell asplasia (PRCA) is a syndrome characterized by anemia, reticulocytopenia, and markedly reduced or absent erythroid progenitor cells in the bone marrow with preservation of the other hematopoietic lineages. It may present as an isolated primary hematological disorder or secondary to parvovirus infection, collagen vascular disease, leukemia, lymphoma, thymoma, solid tumors, treatment with recombinant human erythropoietin or other drugs, and pregnancy. PRCA is acute and self-limiting. One case of PRCA has been published, of a 63-year-old Chinese man with acute liver failure associated with HEV infection, who improved with supportive care [113].

#### Secondary hemophagocytic syndrome

Secondary hemophagocytic syndrome (HPS), sometimes known as the macrophage activation syndrome, is a hyperinflammatory condition, characterized by excessive macrophage function. It is a rare, life-threatening complication of infection, hematological cancer, drug exposure, and autoimmune disease. The most common infectious trigger is EBV, but HIV is increasingly implicated, as well as other infections. There are no validated diagnostic criteria for HPS, but suggestive features include high temperatures, organomegaly, cytopenias and coagulopathy, markedly elevated ferritin levels, hypertriglyceridemia, and hypofibrinogenemia [114]. Four cases of HPS secondary to HEV infection have been published [115–118] (Table 7); high serum ferritin level was noted in all cases. Co-morbidities were observed in three cases (hepatitis A co-infection, splenic lymphoma, and rheumatoid arthritis treated with tocilizumab infusion; these could have played a role in the occurrence of hemophagocytic syndrome, due to known associations). Three patients recovered with supportive treatment, and the fourth died due to fulminant hepatitis.

**Table 7.** Hemophagocytic syndrome secondary to HEV infection

Authors/year	Age/sex	Country	HEV diagnosis/genotype	Co-morbidity	Bone marrow aspirate	Hemoglobin (mg/dL)	Leucocytes (/mm^3^)	Platelets (/mm^3^)	Ferritin ng/mL	Treatment	Outcome
Kamihira 2008 [115]	52/M	Japan	PCR/3	–	–	14.3	2 800	20 000	23 200	Supportive	Recovery
Kaur 2011 [117]	6/F	India	IgM/NT	HAV co-infection, hepatic encephalopathy	HPC	6.2	–	180 000	1 923	Steroids	Death
Brun 2013 [116]	32/M	France	IgM/NT	Splenic lymphoma	Normal	Low	Low	Low	2 452	Supportive	Recovery
Leroy 2005 [118]	33/M	France	IgM, PCR/NT	Rheumatoid arthritis, tocilizumab infusion	HPC	–	–	63 000	8 856	Supportive	Recovery

HPC = hemophagocytosis; NT = not tested

#### Other hematological manifestations

Monoclonal gammopathy of undetermined significance (MGUS), without clinical or laboratory findings suggestive of myeloma or lymphoma, was noted in 17 out of 65 patients (26%) in a recent retrospective study of autochthonous acute and chronic hepatitis E in Cornwall, UK; however, bone marrow biopsy—which allows differentiation between MGUS and monoclonal gammopathy secondary to viral infections—was not performed in these patients [119]. Paraproteinemia disappeared in three out of six patients after a median of 44.5 months with follow-up serum electrophoresis.

One case of HEV-associated severe agranulocytosis was reported in a 70-year-old Spanish patient infected with genotype 3, with fatal outcome despite treatment with granulocyte-colony stimulating factor and a broad-spectrum antibiotic [120].

## Renal manifestations of HEV and cryoglobulinemia

### Renal manifestations

HEV infection has recently been reported to be associated with renal manifestations. A statistically—but not clinically—significant decrease of estimated glomerular filtration rate (eGFR,-5 mL/min) has been described in France in 51 transplant patients during the acute phase of HEV infection genotype 3 [121]. The decrease appeared to be related to HEV, since other causes were ruled out (e.g. acute rejection, infection, modification in immunosuppression regimen). One case of HEV-related acute tubular necrosis has been reported in an immunocompetent patient who was successfully treated with steroids [122]. Two cases of acute renal failure of unknown cause, in association with HEV infection, were reported, one case in a kidney transplant patient who recovered with supportive treatment [123], and a second case in an Indian patient with severe hyperbilirubinemia that responded to hemodialysis [124]. Renal biopsy was not done in either of these cases. Finally, at least eight cases of glomerulonephritis, associated with nephrotic syndrome and/or mixed cryoglobulinemia, have been described [101, 121–126] (Table 8). Types of renal injury included membranoproliferative glomerulonephritis, membranous nephropathy, relapsing IgA nephropathy, and nephroangiosclerosis; seven of these cases occurred in immunosuppressed patients. Immunosuppressant dose reduction or antiviral administration led to complete recovery in three patients and stabilization in one, whereas end-stage renal disease occurred in three patients. It is noteworthy that six out of eight cases of glomerulonephritis were published by the Toulouse group [121, 124, 125] which raises the possibility that other cases may have gone undetected elsewhere. The mechanism of HEV-induced kidney disease could be immune-driven in a manner similar to that with HCV. HEV should be screened for in cases of glomerulonephritis, especially if it is associated with transaminase elevation. Ribavirin can then be used to obtain a rapid viral clearance.

**Table 8.** Renal manifestations associated with HEV infection

Authors/year	Age/sex	Country	HEV diagnosis/genotype	Associated disease	Renal manifestations	Treatment	Outcome
Verschuuren 1997 [122]	34/F	Netherlands	IgM/NT	None	ATN (unknown cause)	Steroids	Recovery
Ali 2001 [101]	38/M	India	IgM/NT	None	MGN	Steroids	Recovery
Kamar 2005 [123]	28/M	France	PCR/NT	Renal transplant	Renal failure (unknown cause), no renal biopsy	Supportive	Recovery
Kamar 2012 [121]	33/M	France	PCR/3f	Renal transplant	MPGN, NS	TAC reduction	Recovery
	26/M	France	PCR/3f	Renal transplant	IGAN relapse, MC II, NS	Ribavirin	Stable
	40/M	France	PCR/3f	Renal transplant	IGAN relapse, MC II, NS	TAC reduction	End stage renal disease end stage renal disease
	24/M	France	PCR/3f	Renal transplant	MPGN, MC III, NS	Rituximab	End stage renal disease
	58/M	France	PCR/3c	Liver transplant	NAS, MC III, NS	PegIFN	
Vikrant 2013 [124]	56/M	India	IgM/NT	Severe hyperbilirubinemia	Renal failure (unknown cause), no renal biopsy	Hemodialysis	Improvement, Sudden CV arrest
Taton 2013 [125]	60/M	France	PCR/3c	Renal transplant	MN, NS	Ribavirin	Recovery
Kamar 2015 [123]	46/M	France	PCR/3f	Renal transplant	MPGN, MC	TAC reduction, ribavirin	Recovery
Del Bello 2015 [126]	46/M	France	PCR/3f	Renal transplant	MPGN	TAC reduction, ribavirin	Recovery

ATN = acute tubular necrosis; CV = cardiovascular; IGAN = IgA nephropathy; MGN = membranous glomerulonephritis; MC = mixed cryoglobulinemia; MN = membranous nephropathy; MPGN = membranoproliferative glomerulonephritis; NAS = nephroangiosclerosis; NS = nephrotic syndrome; NT = not tested; PegIFN = pegylated interferon; TAC = tacrolimus

### Mixed cryoglobulinemia

Mixed cryoglobulinemia has been associated with several viral infections; at least nine viruses have been implicated [127]. HCV chronic infection is recognized as the major cause of mixed cryoglobulinemia, reported in 90% of Italian patients in one series [128], although later studies found wide geographical variations [129]. Some cases of mixed cryoglobulinemia are related to HIV [130], HBV [131] and, less frequently, to HAV [132], as well as other viruses.

Four reports of HEV-related mixed cryoglobulinemia, associated with glomerulonephritis and/or nephrotic syndrome, have been published, with a total of 11 patients [121, 126, 133, 134] (Table 9). In one of these cases, HCV-HEV co-infection was present, and HEV RNA was not tested, which makes this case a probable HEV-related mixed cryoglobulinemia [133]. In the other 10 cases, HEV-related mixed cryoglobulinemia was well documented. In all cases published thus far, the presence of HEV RNA in the cryoprecipitate was not evaluated.

**Table 9.** Mixed cryoglobulinemia associated with HEV infection

Authors/year	No. of cases	Country	Age/sex	Co-morbidity	Cryoglobulinemia		HEV infection		
					Type	Manifestation	Diagnosis/genotype	Treatment	Outcome
Marson 1995 [133]	1	Italy	62/F	HCV co-infection	II	Peripheral neuropathy	IgG/NM	NM	NM
Kamar 2012 [121]	8	France	NM	Solid organ transplantation	II–III	Glomerulonephritis, nephrotic syndrome	PCR/3	PegIFN or ribavirin	Negative PCR 3 month after beginning of treatment, but SVR not reported
Pischke 2014 [134]	1	Germany	35/M	Liver transplant	III	Arthralgia, myalgia, thrombocytopenia	PCR/NM	Steroids	Death (mucositis)
Del Bello 2015 [126]	1	France	46/M	Renal transplant	III	MPGN	PCR/3f	TAC reduction then ribavirin	SVR, recovery

MPGN = membranoproliferative glomerulonephritis; NM = not mentioned; PegIFN = pegylated interferon; TAC = Tacrolimus; SVR = sustained virological response

HEV-related mixed cryoglobulinemia occurred during active infection in 9 cases or after viral clearance in one case [134]. The occurrence of mixed cryoglobulinemia after viral clearance is similar to what has been observed in other extra-hepatic manifestations related to HEV infection. As with other viral infections, HEV could trigger autoimmunity, which could explain the development of extra-hepatic manifestations after viral clearance [135]. All reported patients with mixed cryoglobulinemia (i) were immunosuppressed because of solid organ transplantation, (ii) had chronic hepatitis E with persistent HEV replication for more than 3 months, and (iii) originated from western Europe, where genotype 3 is prevalent, with confirmation of this genotype in nine patients. All patients had type II or III mixed cryoglobulinemia. Antiviral treatment (peginterferon or ribavirin) was given in nine cases. Viral clearance and negativity of cryoglobulinemia were obtained in all patients 3 months after the beginning of antiviral treatment. Similarly, rheumatoid factor, when present, disappeared and the C3 complement component was slightly decreased during antiviral therapy. Immunosuppressive treatment was given in one case (steroids and increase of immunosuppressants) with rapid symptomatic improvement; however, two relapses occurred after reduction of corticosteroids. During the second relapse, the patient developed an acute, fatal episode of severe intestinal mucositis.

HEV infection should be added to the other viral infections causing mixed cryoglobulinemia [136]. Further studies are needed to delineate the frequency of HEV-related mixed cryoglobulinemia, its pathophysiology, and to confirm in a larger cohort of patients the excellent—albeit preliminary—data on antiviral treatment.

### Other possibly immune-mediated manifestations

Six cases of thyroid diseases (three cases of Grave’s disease, one of subclinical hyperthyroidism, one of subacute thyroiditis, and one of painless thyroiditis) have been published [137–141]. It was suggested that HEV might be a trigger for the development of autoimmune thyroiditis [137]. Three cases of HEV-related myocarditis have been published, with full recovery either spontaneously or following treatment with indomethacin or steroids [142–144]. One case of Schöenlein-Henoch purpura, which resolved itself spontaneously after clearance of the virus [145], and another case of *myasthenia gravis,* which resolved itself after treatment with ribavirin and intravenous immunoglobulin [146], have also been reported (Table 10). Further studies are needed to confirm these associations.

**Table 10.** Other possibly autoimmune extra-hepatic manifestations associated with acute HEV infection

Authors/year	Country	Age/sex	HEV diagnosis /genotype	Manifestations	Treatment	Outcome
**Thyroid diseases**						
Hui 2003 [139]	Hong Kong, China	38/M	IgG-IgM/NT	Inactive HBsAg carrier, Grave’s disease, fulminant hepatitis	Lithium, methimazole	Recovery
Kong 2006[138]	South Korea	34/F	IgG-IgM/NT	Subclinical hyperthyroidism	PTU	Recovery
	South Korea	42/M	IgG-IgM/NT	Grave’s disease	intractable to PTU	Recovery
Dumoulin 2012 [137]	Germany	NM/W	IgM/NT	Grave’s disease	Carbimazol, radioiodine	Recovery
Martinez-Artola 2015 [140]	Argentina	45/M	IgG-IgM/3a	Subacute thyroiditis	Supportive	Recovery
Inagaki 2015 [141]	Japan	65/F		Painless thyroiditis, severe hepatitis	Steroid	Recovery
**Myocarditis**						
Goyal 2009 [142]	India	21/M	IgM/NT	Dyspnea, hypotension, acidosis	Ventilation, steroids inotropic support,	Recovery
Dougherty 2012 [143]	USA	50/F	IgM/NT	Chest pain, palpitations, dyspnea	Indomethacin	Recovery
Premkumar 2015 [144]	India	26/M	IgM/NT	Acute kidney injury	Supportive, SLED	Recovery
**Other manifestations**						
Thapa 2010 [145]	India	6/F	IgM/NT	Schöenlein, Henoch purpura	Supportive	Recovery
Belbezier 2014 [146]	France	33/W	RNA/3f	Myasthenia gravis, anti musk+	prostigmine, IVIG, ribavirin	Recovery

IVIG: intravenous immunoglobulin; MuSK: muscle specific kinase; NT: not tested; PTU: propyltuiouracil; SLED: slow low efficiency dialysis

## Conclusion

Numerous extra-hepatic manifestations have been described in patients with HEV infection, mostly as case reports or small case series. Most of these reports were published during the last 5 years, which reflects increased awareness of HEV infection in regions where the disease is not endemic, as well as increased awareness of the extra-hepatic manifestations associated with this infection in general. Acute pancreatitis, neurological disorders with predominantly peripheral nerve involvement, hemolytic anemia due to G6PD deficiency, severe thrombocytopenia, glomerulonephritis, and mixed cryoglobulinemia are the most frequent. For several manifestations, there is a possibility that the association was conjectural. One needs to distinguish between anti-HEV IgM positivity and actual clinical illness resembling hepatitis. Such critical evaluation was not feasible. The other extra-hepatic manifestations are uncommonly reported. They may develop either in acute or chronic infection, and during active infection or after clearance of HEV infection. Unless a diagnosis of HEV infection is specifically sought, the diagnosis will be missed because the clinical presentations overlap with many other disorders. We anticipate that more extra-hepatic manifestations of HEV will be reported in the future and that a greater understanding of their immuno-pathogenesis and treatment will evolve.
